# After All, Only Millions?

**DOI:** 10.1128/mBio.00999-16

**Published:** 2016-07-05

**Authors:** Rudolf Amann, Ramon Rosselló-Móra

**Affiliations:** a Max Planck Institute for Marine Microbiology, Bremen, Germany; b IMEDEA (CSIC-UIB), Esporles, Spain

## Abstract

An update on the census of species of *Archaea* and *Bacteria* published recently in mBio (P. D. Schloss, R. A. Girard, T. Martin, J. Edwards, and J. C. Thrash, mBio 7:e00201-16, 2016, http://dx.doi.org/10.1128/mBio.00201-16) showed again that, despite ever-increasing sequencing efforts, the PCR-based retrieval of 16S rRNA genes is approaching saturation. On average, 95% of the genes analyzed today are identical to those present in public databases, with rarefaction analysis indicating that about one-third of the bacterial and archaeal diversity has already been covered. Therefore, despite estimates of up to 10^12^ microbial species, the option should be considered that the census of *Archaea* and *Bacteria* on planet Earth might yield only millions of species after all.

## COMMENTARY

We live in a world of numbers. Who would not prefer a net worth of billions rather than millions or thousands? The census of *Bacteria* and *Archaea* is also a numbers game, yet one of great importance for microbiology and microbiologists. How many species of prokaryotes can we put forward to demonstrate that our discipline is on par with botany or zoology? A publication in mBio provided a meta-analysis of datasets (1) which seems to starkly disagree with a recent prediction made on the basis of scaling laws that planet Earth is home to a trillion (10^12^) microbial species (2).

Let us briefly recapitulate how census estimates have changed over the past decades. The implementation of the Bacteriological Code in 1980 created official rules for the nomenclature of species. Consequently, the numbers of valid prokaryotic species and genera published in the Approval List went down to about 1,800 and 300, respectively, from the previous list of over 30,000 names, most of which were proven to be synonyms (3). Ever since, microbial taxonomists have isolated, characterized, and described several hundred new species per year, with the number of validly named species of *Bacteria* and *Archaea* expected to surpass 13,000 in 2016. The radical restart of microbial taxonomy has been enforced by methods of molecular biology, with the most important method being comparative sequence analysis of the small-subunit rRNA, also known as 16S rRNA. In an epochal discovery, Woese and Fox used this method to demonstrate the deep dichotomy of two fully independent prokaryotic domains, now known as *Bacteria* and *Archaea* (4). In the 1980s, microbiologists estimated the number of species that could be isolated and described in the tens of thousands, while acknowledging the difficulty and the time that would be required to culture and characterize new isolates.

This changed dramatically on 3 May 1990 with two back-to-back publications in *Nature* (5, 6). In both, the authors had skipped cultivation and made use of a targeted PCR-based retrieval of partial 16S rRNA genes from nucleic acids extracted from their respective habitats. The microbial world visible through this new window was quite different, indeed, from what we knew before. Twelve 16S rRNA clones originating from Sargasso Sea surface ocean bacterioplankton were found to contain seven unique sequence types, none matching a known species (5). The result was essentially the same when 16S rRNA was retrieved from the microbial mat of the thermal Octopus Spring in Yellowstone National Park. All eight sequence types detected in 15 clones were newly discovered (6). Throughout the 1990s, the discovery rates remained high. Accordingly, the estimates for the prokaryotic species census skyrocketed to millions (7) or even billions (8). It was evident that cloning and Sanger sequencing would not suffice to bring these estimates to a test. However, when next-generation sequencing became available a decade ago, the promise was that it was possible not only to describe the “rare biosphere” (9) but also to increase the numbers in the species census of *Bacteria* and *Archaea* by a quantum leap.

Now that hundreds of millions of 16S rRNA gene sequences have been deposited in public databases, the data indicate that this census might not yield numbers very different from those few millions of animal, plant, fungus, and protist species expected to exist on planet Earth (10). In their *mBio* study, Schloss et al. (1) used rarefaction to analyze 1,411,234 near-full-length bacterial and 53,546 archaeal 16S RNA gene sequences released in July 2015 by the curated database project SILVA (11). One central result is that the discovery rate is down to 5%. Today, on average, 95 of 100 newly retrieved 16S rRNA genes are known, differing strikingly from the “all-new times” of the 1990s. The slopes of rarefaction curves indicate that the coverage of operational taxonomic units defined on the basis of a 3% sequence distance cutoff approaches 30% for species of *Bacteria*, with the coverage of *Archaea* being close to 40%. This corroborates estimates published in 2014 (12) which indicated that the PCR-based census will approach saturation within a few years. Acknowledging that 16S rRNA sequences are too conserved to be a good proxy for species numbers but give robust results on higher taxonomic levels such as genus (coinciding with a sequence distance of about 5%), the number of bacterial genera could plateau at 200,000 and that of *Archaea* at 5,000. After all, it seems that the prokaryotic species census might ultimately stop in the lower millions rather than in the billions.

Many more aspects of this census update by Schloss and colleagues are worth considering. We highlight the following four. First, next-generation sequencing has brought us not only high throughput but also short sequence lengths. A high-quality census clearly requires near-full-length 16S rRNA sequencing. Second, next-generation sequencing required migration to PCR primers amplifying only parts of the 16S rRNA genes, such as the V4 variable region. Schloss and colleagues used a taxonomic breakdown of sequences submitted to the databases before and after 2006 to confirm that primer sets differ in their representation of diversity. Third, the authors showed that the sequencing effort of the past decade was focused on the human microbiome with its rather limited diversity. An Earth microbiome project coordinating an equally large effort focused on the *Bacteria* and *Archaea* living in soils, sediments, and aquatic and extreme environments will fix this distortion. Finally, the authors provided us with first data on what additional diversity had been accessed by new technology, independently of PCR. Of the few hundred single-cell genomes of *Bacteria* and *Archaea*, less than 10% were newly identified, suggesting that without prior sorting this method would mostly report the abundant clades known from previous studies. In contrast, 85% of the several hundred mostly bacterial sequences assembled from metagenomes by the EMIRGE algorithm had not been recovered before. Although the error rate may be high in assembling 16S rRNA genes with their many highly conserved regions, PCR-independent sequence retrieval will add important data to the census, in particular, when next-next-generation sequencing is providing long reads with very low error rates. Future efforts focused on finishing the census of *Bacteria* and *Archaea* need certainly to include PCR-independent technologies, much as the big step of 1990 required culture independence.

In a numbers world, millions are less than billions or trillions. The study of Schloss et al. provides further evidence that the species census of *Bacteria* and *Archaea* might stop at a few millions. The tail observed in rank abundance curves could be not so long, after all. Is the “rare biosphere,” as observed in essentially all microbial diversity studies, just the result of stochastic dispersal of persistent *Bacteria* and *Archaea* from a large, yet limited pool? In yet-to-be-disclosed habitats, the “rare biosphere” microorganisms might be abundant. Should this lower count make microbiologists less confident? Of course not! Many of the species of *Bacteria* and *Archaea* that were discovered in the past decades and yet remain uncharacterized are very divergent from all the species that we know. Yarza et al. (12) have made an effort to unite the classifications of cultured and uncultured prokaryotes. In doing so, they have deduced 16S rRNA-based distance thresholds from the Living Tree Project (13), only to show that there are likely more than 1,000 phylum-level clades that lack any cultured representative. There is still much biology to be discovered in these candidate phyla, although biochemical diversity might not scale with phylogenetic diversity. Last, but not least, we should get rid of the nasty remark that “bacterial and archaeal diversity is so incomprehensibly vast that it is just not worth describing.” The good news might rather be that the number of microbial taxa represents an order of magnitude well within reach of today’s technologies. Let us use our creative minds to access as-yet-undisclosed microbial habitats with sampling and analysis strategies that focus on the new.
